# Extremely Rare Case of a Giant Paratubal Cyst, Coexisting with a Mucinous Cystadenoma, Surgically Treated Through Laparoscopy—A Case Report and Review of the Literature

**DOI:** 10.3390/reports8030106

**Published:** 2025-07-14

**Authors:** Tudor Andrei Butureanu, Ana-Maria Apetrei, Ioana Pavaleanu, Ana-Maria Haliciu, Razvan Socolov, Raluca Balan

**Affiliations:** 1 Department of Mother and Child, “Gr. T. Popa” University of Medicine and Pharmacy, 700115 Iasi, Romaniaanna_cefalan@yahoo.com (A.-M.H.); socolov.razvan@gmail.com (R.S.); 2 “Elena Doamna” University Hospital of Obstetrics and Gynecology, 700115 Iasi, Romania; raluca12usa@yahoo.com; 3 Department of Morphofunctional Sciences I, “Gr. T. Popa” University of Medicine and Pharmacy, 700115 Iasi, Romania

**Keywords:** laparoscopy, minimally invasive surgery, adnexal mass, tumor, giant

## Abstract

**Background and Clinical Significance**: A paratubal cyst, which makes up about 10% of all adnexal masses, is a specific type of adnexal cyst that develops from the mesothelium in the broad ligament located between the fallopian tube and the ovary. Interestingly, the majority of paratubal cyst cases are initially misidentified as ovarian cysts, with suspicion arising in only 1 out of every 15 patients before undergoing surgery. **Case Presentation**: We report a case of a giant paratubal cyst mimicking an ovarian cyst in a 21-year-old woman supported by some representative images along with a literature review. The cyst’s therapeutic management was surgical removal of the adnexa and the final postoperative histopathological diagnosis was that of a benign paratubal cyst. **Conclusions**: This case highlights the need to include a paratubal cyst in the differential diagnosis of pelvic masses, especially in women of reproductive age. To the best of our knowledge, this represents the largest paratubal cyst reported in the literature to date, based on overall dimensions and the highest recorded volume of aspirated fluid, successfully managed via laparoscopy. A further notable aspect of this case is the coexistence of the giant paratubal cyst with an ovarian mucinous cystadenoma.

## 1. Introduction and Clinical Significance

A paratubal cyst is a form of adnexal cyst that develops from the mesothelium within the broad ligament connecting the fallopian tube and the ovary. Paratubal cysts are generally asymptomatic and reduced in size and account for about 10% of all adnexal masses [1]. A giant paratubal cyst is rare, and complications such as torsion could be a cause of abdominal pain.

A paratubal cyst is a fluid-filled sac lined by ciliated tubal-type epithelium that typically develops in close proximity to the ovary and is often connected to the fallopian tube. It is considered that the origin of these cysts is probably in the Müllerian (paramesonephric) and Wolffian (mesonephric) ducts, embryological tubular structures that coexist in the early stages of embryonic development and that are further remodelled in order to give rise to the genital organs. During embryological development, these ducts, or portions of these ducts, can invaginate and form cycstic dilatations, leading to the formation of paratubal or paraovarian cysts [2].

Other hypotheses suggest cystic changes in endosalpingiosis or mesothelial transformation toward Müllerian tissue [1,3]. Regarding the incidence of paratubal cysts, the literature reports a 7.3% incidence in children and adolescents aged between 0 to 19 years old [4]. The real incidence of such cystic structures is difficult to assess, as these structures frequently do not cause any noticeable symptoms and are usually discovered incidentally during other pelvic examinations or surgical procedures [5].

One clinical context that leads to this diagnosis is adnexal torsion caused by the large volume of the paratubal cyst, a complication that requires prompt surgical treatment. Other unfavorable evolutions could be neoplastic development, supporting previous reports that demonstrate the possibility of malignant progression of paratubal or paraovarian cysts in 2 to 3% of cases [3,6]. Borderline transformation is also possible in these types of cysts [7], resulting in abnormal proliferation of the epithelial lining accompanied by cellular atypia, but without stromal invasion, morphological features that characterize the borderline malignant transformation [8,9]. Another complication of paratubal cysts is recurrence, as described by Magistrado et al., with this eventuality being difficult to anticipate or predict, which makes patient counseling more difficult [10].

When symptomatic, they are usually accompanied by abdominal pressure due to their large volume, or acute pain, when complications such as torsion or rupture occur [11]. The potential transformation of paratubal cysts into giant cysts has been mentioned in various published case reports [11,12,13,14,15], but previous therapeutic approaches have included classical surgical interventions involving laparotomy or modified laparotomy, even though most clinicians agree that minimally invasive techniques should be implemented when they are technically possible and the surgeon’s experience allows it. We report the case of a 21-year-old woman with a giant paratubal cyst mimicking an ovarian cyst, which was treated through laparoscopy, along with a literature review, searching the PubMed, ScienceDirect, and Google Scholar databases in order to retrieve studies or case reports about “giant paratubal cysts” and “minimally invasive surgery in large adnexal masses”. To the best of our knowledge, this is the biggest paratubal twisted cyst reported so far in the literature treated with such a therapeutic approach.

## 2. Case Presentation

**Figure 1:**
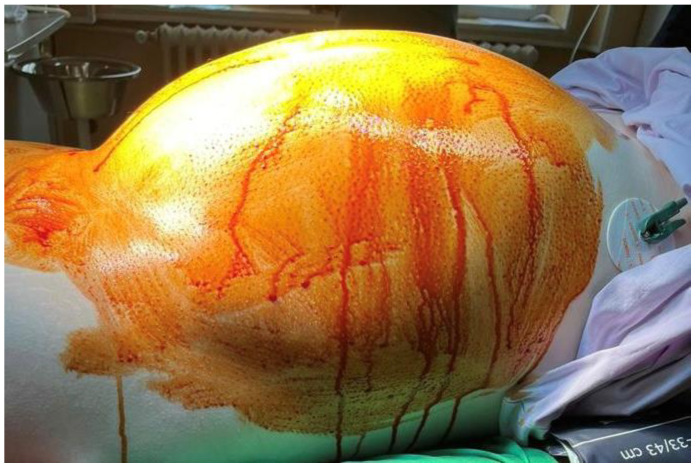
Patient with a perioperatively abdominal mass.

**Figure 2:**
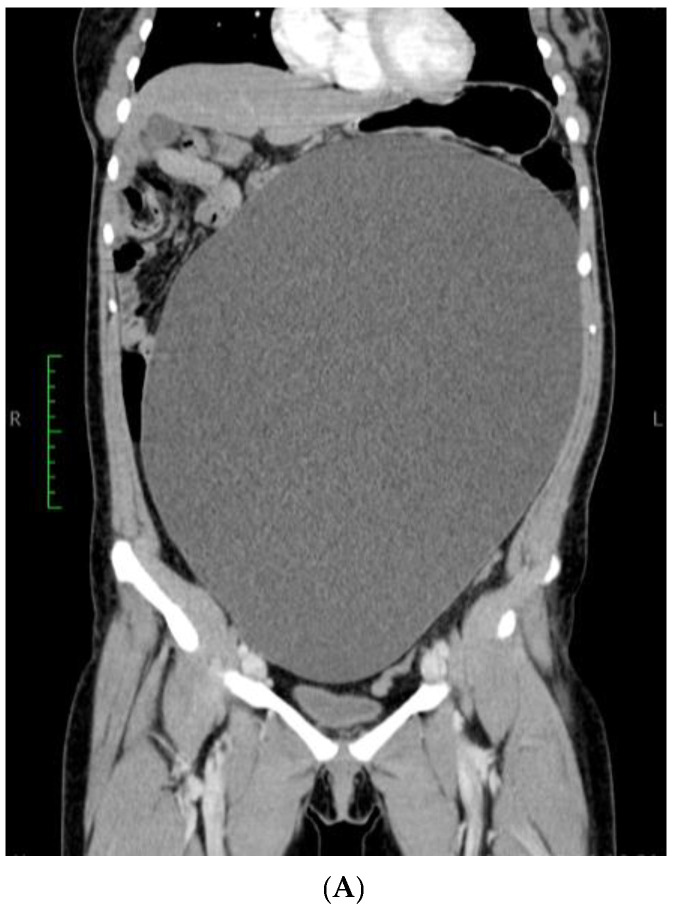

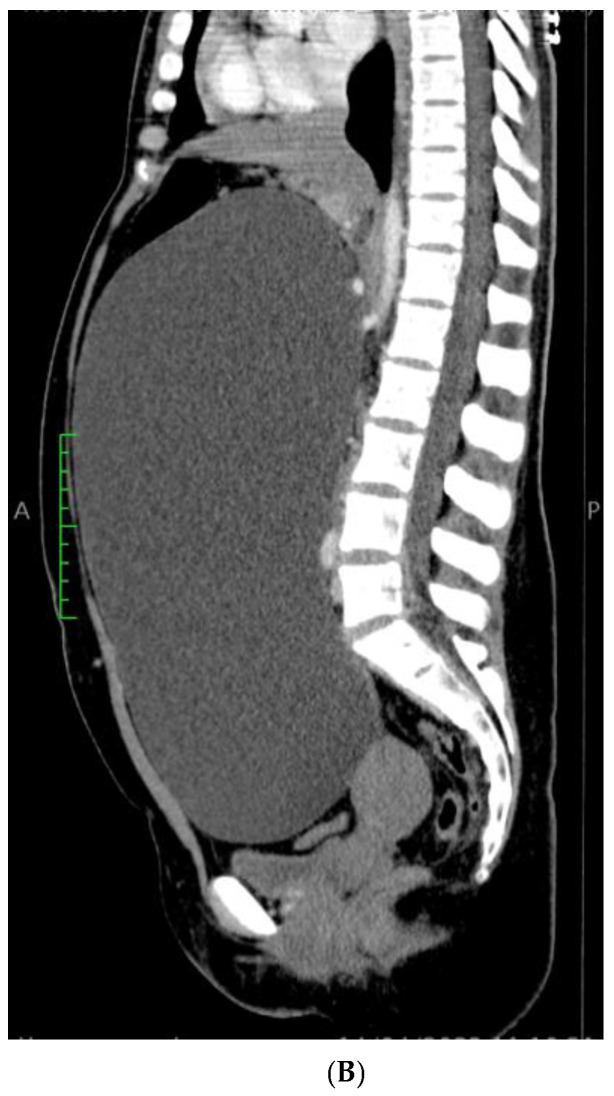
CT Scan: Gigantic ovarian cyst of 360/300/150 mm. (**A**) Sagittal section and (**B**) coronal section.

A 21-year-old, nulliparous, virgin woman presented to the Gastroenterology Department with the acute onset of upper abdominal pain but without any other symptoms such as chronic pain, abdominal distension, weight gain, or intra-abdominal pressure. She is a White/Caucasian woman from a rural environment with no medical history, and she lacked family history data. Moreover, the patient did not have any urinary or bowel symptoms. Her menstrual cycle was regular and normal, with her last menstrual period 3 weeks prior to hospital admission. Her full blood count was normal, and the microscopic examination of the urine sediment and urine culture did not show evidence of urinary tract infection. A urine pregnancy test was not performed due to the fact that she had never had intercourse. She had not received any treatment prior to the gynecological examination. The patient’s weight was 79 kg. Abdominal inspection mimicked a term pregnancy (Figure 1), and bimanual pelvic examination was not performed. Abdominal ultrasound performed at another medical facility revealed a simple cystic mass that was difficult to measure. No Doppler flow was detected, no septa nor papillary projections were noted, and there was no ascites. The left ovary appeared normal with only a 20 mm follicle. CA125, CEA, and CA 19-9 levels were within normal limits. The patient did not have immediate access to Magnetic Resonance Imaging (MRI), so she was referred for computer tomography (CT) examination which revealed a gigantic abdominal cystic mass of 150/300/360 mm (antero-posterior/transversal/cranio-caudal), most probably of ovarian origin with a thin wall with no septum or calcification that moves the retroperitoneal structures and compresses the bowel. The right ovary could not be observed. The left ovary measured 39/26 mm and presented few follicles, the largest being 26 mm. There were no abnormalities in the uterus, intraperitoneal fluid was absent, and no abnormal lymph nodes were identified. All other structures were within normal limits (Figure 2A,B).

**Figure 3:**
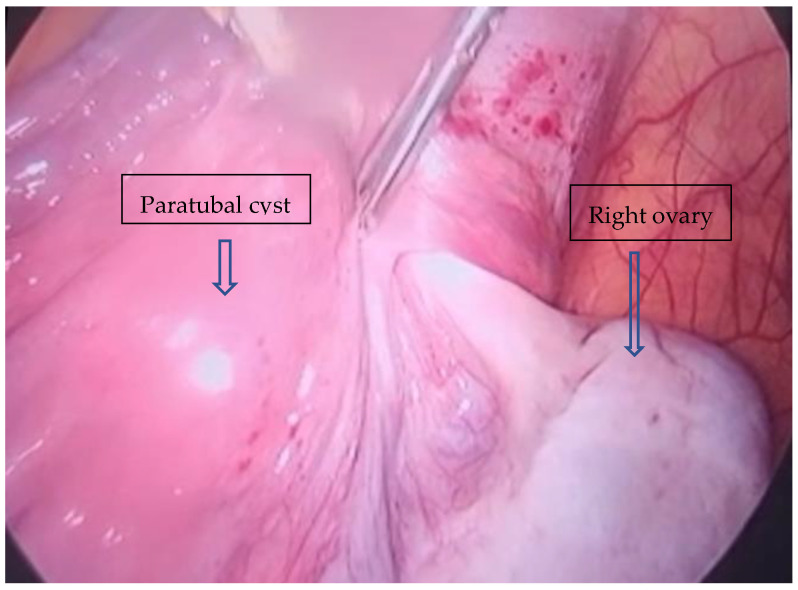
Intraoperatively discovered adnexal torsion.

**Figure 4:**
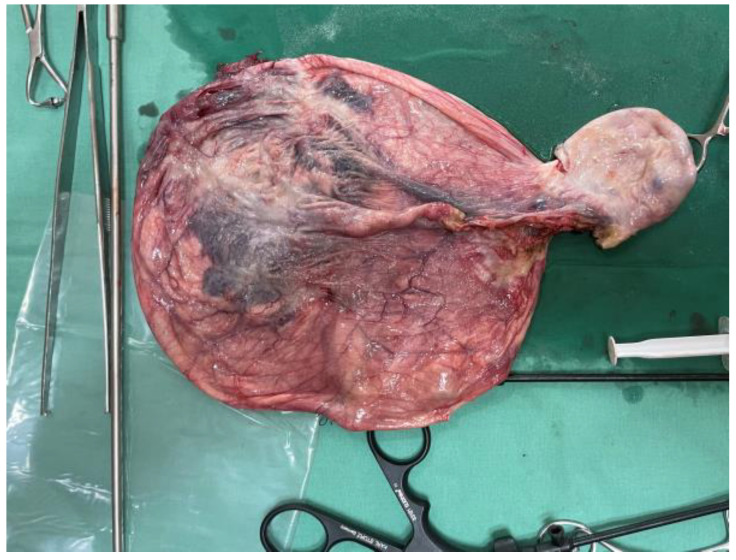
Right laparoscopic adnexectomy.

After tumor drainage through the umbilical open laparoscopy incision, a laparoscopic adnexectomy was performed. As we commonly employ the Hasson open laparoscopy technique, a lateral umbilical incision was made, through which we aspired 10.5 L of cyst fluid without spillage. Thereafter, a suture of the tumoral incision was performed to avoid any further spillage of the remaining fluid. Mass drainage was without incident, with no compartment syndrome after evacuation. A partially twisted, large, and well-vascularized right-sided paraovarian cyst (Figure 3) was revealed. After careful inspection of the abdomen, a right adnexectomy was performed using bipolar forceps and scissors (Figure 4).

The fallopian tube was stretched along the surface of the cyst. Although cystectomy was technically difficult due to the extent of the cystic walls, the adnexa was extracted through the lateral umbilical incision. Abdominal cavity washing with cytological examination was also performed.

The initial intraoperative histopathological diagnosis revealed a benign paratubal cyst.

The final histopathological report described two macroscopically cystic masses, one of 12/11/8.5 cm after intraoperative drainage, without any papillary projections, with a fallopian tube of 15 cm, in close proximity to the mass, and another one of 6.8/3.8/3.5 cm without any vegetations.

**Figure 5:**
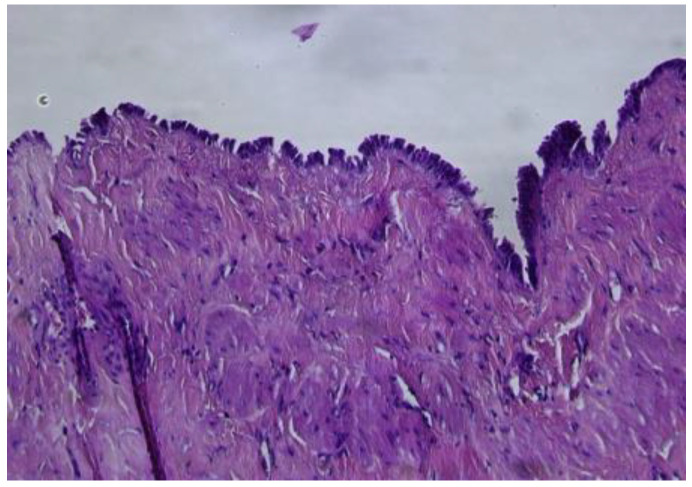
Paratubal cyst lined by pseudostratified and simple columnar epithelium (HE ×20).

The histopathological microscopic assessment of the first structure revealed a cyst wall lined by a ciliated tubal-type epithelium (Figure 5), with endosalpingeal focal papillary projections.

**Figure 6:**
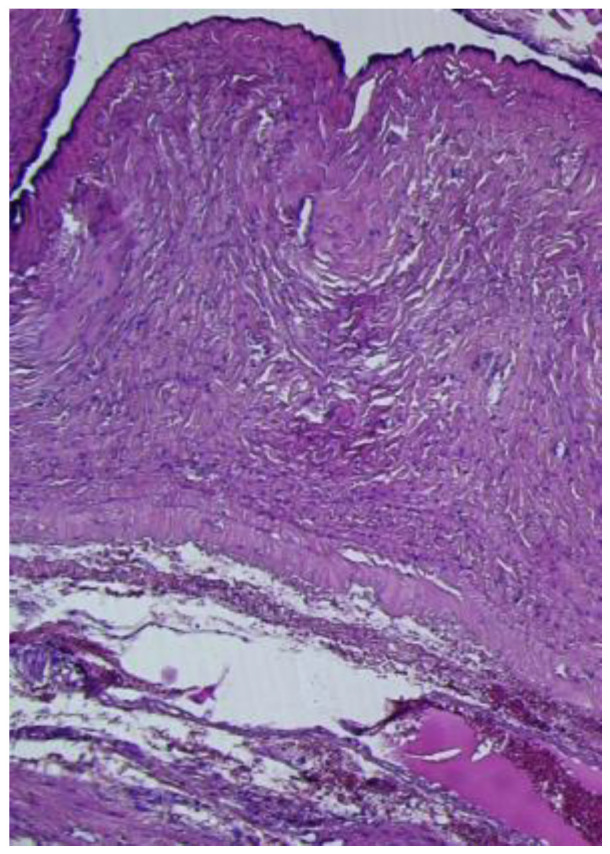
Paratubal cyst, lined by a simple, flat, cuboidal to columnar epithelium, in morphological continuity with the fallopian tube wall (HE ×10).

The cyst had a morphological connection with the fallopian tube (Figure 6), which presented a mucosa lined by a simple columnar epithelium with focal stratification.

**Figure 7:**
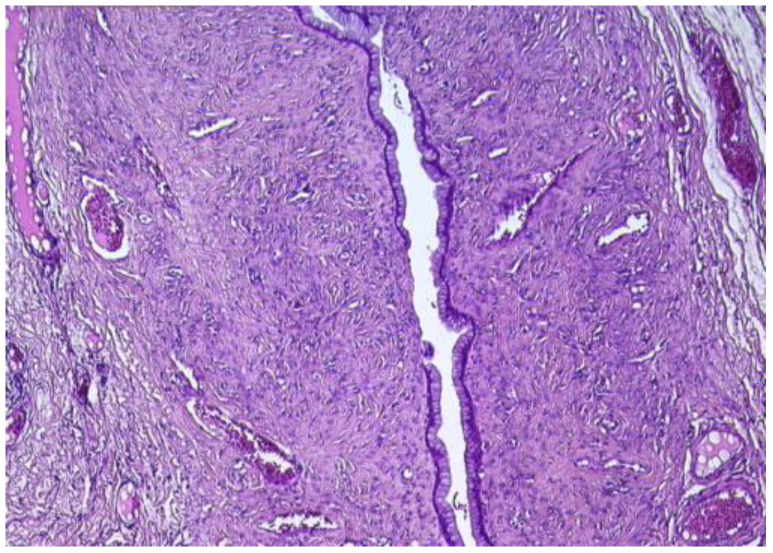
Ovarian mucinous cystadenoma, lined by simple, mucinous columnar epithelium (HE ×10).

In the ovary, the microscopic exam revealed the second cyst, which was lined by a simple mucinous columnar epithelium, with focal glandular structures (Figure 7).

The epithelium presented isolated cuboidal to columnar cells with intracytoplasmic mucin and minor cytology atypia. The subjacent ovarian stroma presented luteinized areas, predominantly periglandular, accompanied by lymphoplasmacytic infiltrate, as well as rare calcifications. The remaining ovarian stroma presented follicles in different evolutive stages, atretic follicles, and a follicular cyst. Vascular ectasia was also observed.

## 3. Discussion

To the best of our knowledge, this is the biggest paratubal cyst or adnexal mass found in the literature so far, with all measurements accounted for and having the highest volume of aspired fluid, to be managed through laparoscopy. Another particularity of our case is the association between the giant paratubal cyst and an ovarian mucinous cystadenoma.

Paratubal cysts are common tumor-like lesions, usually small in size. They are also called paraovarian cysts.

It is considered that paratubal cysts originate from the mesothelium or represent remnants of Wolffian or Müllerian ducts.

In the general population, the incidence is found to be around 3%, with the majority of these tumors occurring at reproductive age, most frequently between the third to fifth decades [12]. There are also similar reported cases at extreme ages, like childhood, adolescence, or postmenopause [14].

Most paratubal cysts are diagnosed intraoperatively, with only 1 in 15 patients being diagnosed prior to surgery [5]. Thus, ultrasonography represents a reliable method for diagnosing and locating paratubal cysts [15]. Ultrasonographic differential diagnosis includes a peritoneal inclusion cyst, hydrosalpinx, or an ovarian cyst [14]. In this regard, an intraoperative finding of vascularization crossing over the cystic tumor represents a pathognomonic sign of a paratubal cyst, helping in differentiation from an ovarian cyst [11].

Detecting borderline ovarian tumors before surgery is crucial, particularly for young women who wish to preserve their fertility. This approach allows for the possibility of less aggressive, fertility-preserving surgery instead of the more radical procedures often required for malignant tumors. Ludovisi et al. [16] outlined the characteristics of Serous Surface Papillary Borderline Ovarian Tumors, which are typically found on the surface of the ovary and appear as irregular solid masses surrounding healthy ovarian tissue. In one study, Moro et al. [17] defined the clinical and ultrasound characteristics of various subclasses of malignant and borderline ovarian tumors. They characterized borderline ovarian tumors as either unilocular–solid or multilocular–solid with solid papillary projections, noting an ultrasound appearance that overlaps between borderline ovarian tumors and non-invasive, low-grade serous ovarian carcinoma. In the same study, Moro et al. showed that in 81.3% of borderline ovarian tumors, papillary projections were present, with more than three projections in most cases [17].

When evaluating the risk of malignancy in an ovarian mass, numerous scoring systems based on ultrasound are available. The IOTA group has introduced two distinct models for predicting malignancy risk: the ultrasound Simple Rules and the ADNEX model [18].

ADNEX without CA125 and the two-step strategy can effectively discriminate benign and malignant masses, but the two-step strategy has higher utility in practice at risk of malignancy thresholds <3%. For the IOTA two-step strategy, the first step is to check whether any of the four Benign Descriptors (BDs) apply to the mass. If none of the BDs apply, ADNEX is used to calculate the risk of malignancy [19].

The Society of Radiologists in Ultrasound, through a consensus statement, recommends that cysts exceeding 7 cm in size should undergo further examination with MRI, regardless of the patient’s age [20].

Identifying a healthy ovary close to but distinct from the cyst is a key radiological indication for diagnosing paraovarian cysts. While transabdominal ultrasound can also detect these cysts, MRI becomes useful if the ipsilateral ovary with the cyst is not clearly visible separately. Moreover, MRI is the preferred method for a more detailed examination of complex paraovarian masses or when there are suspicions of neoplasia, as it provides a clearer outline of the mass in the pelvis and assesses the lesion’s blood supply. However, the high cost of MRI restricts its use, especially in low-resource settings like ours [20]. Differences in imaging methods describing paratubal cysts are shown in Table 1.

Kishimoto et al. have described the MRI characteristics of these cysts. A dilated tube indicates a tubular structure, while an endometrioma appears multifocal and exhibits low signal intensity on T2-weighted MRI images [20]. Paraovarian cysts display low signal intensity on T1-weighted images and uniformly high intensity on T2-weighted images. If a cyst is complicated by hemorrhage, it shows high intensity on T1-weighted images [20].

These cysts usually do not cause any symptoms and often are not diagnosed unless they cause pain, are diagnosed incidentally upon imaging, or are discovered during laparoscopic surgery or laparotomy. Most of these cysts are small (between 1 and 8 cm, rarely more than 20 cm), with their size being correlated with obesity [21]. In such cases, computer tomography (CT) can be helpful, as it can exclude other causes of acute lower abdominal pain, such as appendicitis, ruptured ovarian cyst, ectopic pregnancy, acute ureteric colic, or pelvic inflammatory disease [14]. Enlargement, hemorrhage, adnexal torsion, or rupture of paratubal cysts have been reported, but these are rare causes of acute lower abdominal pain, nausea, and vomiting [14,15]. Since these cysts lack a pedicle, if they undergo torsion, this frequently involves the ovary, the fallopian tube, and the infundibulopelvic ligament [20,21,22,23]. The incidence of torsion among patients with paraovarian cysts ranges from 2.1 to 16% [14,24,25]. In the event of complications, the diagnosis must be made quickly in order to preserve ovarian function [14].

Gross inspection of paratubal or paraovarian cysts reveals simple cysts, filled with clear fluid and located near the fallopian tube. Histologically, paratubal cysts have many similarities with ovarian serous cystadenomas, but the paratubal cyst wall frequently contains smooth muscle, thus helping in distinguishing them from ovarian cysts [1,9]. Moreover, these cysts are lined by ciliated tubal-type epithelium, with them sometimes being able to present focal papillary projections. The macroscopic and microscopic aspects of our reported paratubal cyst are consistent with the existing literature data. When papillary projections are ultrasonographically diagnosed, an intraoperative pathological examination with frozen sections should be performed in order to confirm the malignancy status of the paratubal cyst [12]. In our case, the internal cyst wall showed a smooth lining after the intraoperative frozen section examination was performed.

The histopathological differential diagnosis takes into consideration hydrosalpinx, an endometriotic cyst, or a serous cystadenoma. Consistent with hydrosalpinx is a dilated tubal lumen with attenuated mucosal folds. Although having similar histology, a serous cystadenoma should be larger than 1 cm in size. An endometriotic cyst is lined by endometrial-type epithelium, with or without gland-like structures, surrounded by endometrial stroma, which can present hemosiderin-laden macrophages [9].

Although paratubal cysts are usually benign, they rarely give rise to serous borderline tumors or even undergo malignant transformation [15,26].

Because of the rarity of malignant paratubal or paraovarian cysts, the assessment of CA 125 levels does not represent a reliable parameter in predicting the malignant transformation of a paratubal cyst [26].

The low incidence of paratubal cysts has led to a lack of standard consensus on their management [26]. In this regard, some paratubal cysts are removed laparoscopically, while several others are removed via laparotomy. There are no standard criteria for laparoscopy or laparotomy.

Experience plays a crucial role, and the tumor size could be a limiting factor [1]. The age of the patient, the patient’s preference, as she chose laparoscopy instead of the classical approach, and the lack of malignancy suspicion were all reasons to opt for the laparoscopic therapeutic approach in our case. Intraoperative management differs depending on whether the approach involves cystectomy, cystectomy combined with salpingectomy, or adnexectomy [26]. We opted for a right adnexectomy due to the presence of a second ovarian cyst. For young patients, cystectomy is preferred [15]. Moreover, if fertility sparing is still necessary, conservative surgery for the preservation of the tubes and ovaries should be the first therapeutic approach for paratubal cysts [12].

## 4. Conclusions

This reported case underscores the importance of considering a paratubal cyst as part of potential diagnoses when dealing with pelvic masses, particularly in individuals of reproductive age. A substantial paratubal cyst can resemble a measurable ovarian cyst both before and during surgery. A thorough examination during the surgical procedure to confirm the presence of a paratubal cyst is crucial. When managing paratubal cysts, the primary approach should involve cystectomy. Laparoscopy should be favored, considering that most of these masses occur at reproductive age and that most of them are benign.

This is the biggest paratubal twisted cyst reported thus far in the literature to be treated through laparoscopy and with the largest volume of liquid.

## Figures and Tables

**Table 1:** Imaging characteristics of paratubal cysts.

Imaging Modality	Characteristic	Description
Ultrasonography (US)	Location	Adjacent to the ovary and fallopian tube; may mimic an ovarian cyst.
	Shape and Margins	Round or oval; thin-walled; well-defined.
	Internal Content	Anechoic (clear fluid); no internal echoes unless complicated.
	Mobility	Mobile, separate from the ovary; probe pressure may help differentiate.
	Doppler Flow	No internal vascularity in simple cysts.
MRI	Signal Characteristics (T1/T2)	T1: Hypointense; T2: Hyperintense, consistent with simple fluid.
	Wall Characteristics	Thin-walled, non-enhancing; absence of solid components.
	Ovarian Differentiation	Normal ovary seen separately; cyst is extraovarian.
	Post-contrast Enhancement	No enhancement unless complicated (e.g., hemorrhagic cyst).
Computed Tomography (CT)	Density	Hypodense, fluid-filled; attenuation similar to water.
	Wall Appearance	Thin wall; no calcifications; well-circumscribed.
	Contrast Enhancement	No enhancement unless complicated or inflamed.
	Relation to Other Structures	Clearly separate from the ovary; usually near the fimbrial end of the fallopian tube.

## Data Availability

The original data presented in the study are included in the article, further inquiries can be directed to the corresponding authors.
